# Associations between psychosocial risk factors, and changes in substance dependence and psychosocial functioning, during engagement with digital cognitive behavioral therapy for methamphetamine use: use of 'Breaking Free from Substance Abuse' by incarcerated people during the COVID-19 pandemic

**DOI:** 10.1186/s40352-022-00190-w

**Published:** 2022-09-07

**Authors:** Sarah Elison-Davies, Jamie Newsome, Andrew Jones, Glyn Davies, Jonathan Ward

**Affiliations:** 1 LifeWorks, Manchester Science Park, Manchester, UK; 2 REFORM Alliance, New York City, New York USA; 3 Division of Population Health, Health Services Research and Primary Care, University of Manchester, Manchester, UK

**Keywords:** Methamphetamine, Prisons, Digital, Treatment, Cognitive behavioral therapy, Breaking Free from Substance Abuse

## Abstract

**Background:**

Methamphetamine use can be associated with involvement with correctional services and incarceration. Traditionally, treatments for methamphetamine use have been delivered in-person – however, lockdowns initiated during the COVID-19 pandemic significantly reduced access to such in-person support in prisons. Therefore, in May 2020 a digital cognitive-behavioral therapy (CBT) program for substance use disorders - 'Breaking Free from Substance Abuse' - was made available across prisons in Ohio in order to meet this treatment gap. This represents the first time this digital CBT intervention has been made widely available to incarcerated people residing in prisons or jails in the United States (US). This was a within-subjects study using data from 2187 Ohio prison residents who engaged with this digital CBT program to address their methamphetamine use.

**Results:**

Participants reported multiple psychosocial risk factors, including moderate to severe substance dependence, depression and anxiety; interpersonal conflict; aggressive behavior; paranoia; and difficulties with work, education and accommodation. Significant reductions in substance dependence, depression/anxiety and biopsychosocial impairment, and improvements in quality of life, were identified in the sample. Risk factors were associated with less positive outcomes, specifically interpersonal conflict and poor mental health. Completion of specific components of the program were associated with more positive outcomes – a dose response was also identified.

**Conclusions:**

Digital CBT can be delivered in secure US correctional settings and may help to fill unmet needs for in-person treatment. Specifically, this digital CBT program may support incarcerated individuals to address methamphetamine use, with outcomes being associated with psychosocial risk factors and program engagement.

## Background

During the COVID-19 pandemic in-person substance use disorder (SUD) treatment was limited, with many services moving to digital and telehealth delivery (Monaghesh & Hajizadeh 2020). However, delivering technology-mediated treatment in secure correctional settings is challenging – despite this, there have been examples of correctional services working innovatively to meet the needs of their populations during the pandemic. This study therefore reports data from prison residents across Ohio who accessed digital cognitive behavioral therapy (CBT) for SUD during the pandemic. This study focuses on methamphetamine-involved residents given the links between use of the drug and involvement with correctional services.

### The links between methamphetamine use and involvement in correctional services

Prevalence of methamphetamine use amongst people in the United States (US) in 2019 was 1.2 million for past-month use (Center for Behavioral Health Statistics and Quality 2020). Methamphetamine use is associated with an unstable lifestyle and psychosocial risk factors that are linked to involvement in correctional services (Cumming et al. 2020), including mental health disorders such as psychosis (Chiang et al. 2019), homelessness (Jones et al. 2020), financial difficulties (McKetin et al. 2020) and interpersonal conflict (Maltman et al. 2020). These factors may increase likelihood of both acquisitive (Goldsmid & Willis 2016) and violent crimes (Liu et al. 2017).

Mental health and other psychosocial difficulties (e.g. homelessness), SUD and offending interact in multiple ways (Hamilton 2014). The “self-medication” hypothesis (Robinson et al. 2011) suggests that some people use substances as a means of coping with their mental health difficulties, or conversely, substance use may directly cause, or exacerbate, pre-existing mental health difficulties (e.g. Boden & Fergusson 2011). Alternatively there may be some other common factor that increases vulnerability to mental health and substance use issues, such as chronic stress (Brady & Sinha 2005) or certain personality traits (Kotov et al. 2010). Substance use and mental health difficulties may start in late childhood and early adolescence (Deas & Brown 2006), often alongside significant childhood adversity (Johnson et al. 2006), with these difficulties then contributing to an increased risk of offending in adulthood (Wiesner et al. 2005).

*Psychosocial* risk factors are found in models describing *criminogenic* risk factors, one of the most influential being the ‘risk-need-responsivity’ model (RNR: Andrews et al. 2006). Risk factors identified within the model include family and/or marital issues, difficulties at school or work, leisure or recreation issues, and substance abuse, in addition to anti-social behaviours, cognitions, personality patterns, and associates. These risk factors provide targets for assessment and intervention in populations involved in correctional services – higher-risk offenders may experience greater reductions in recidivism from RNR-informed interventions compared to lower-risk offenders (Bonta & Andrews 2007).

### Treatment for methamphetamine use and the impact of the COVID-19 pandemic

Methamphetamine-involved offenders have unique treatment needs – use of the drug is often associated with cognitive impairments (Potvin et al. 2018) and risk-taking and erratic behaviors (Homer et al. 2008), and people who use the drug often experience complex mental health issues including psychosis (Cumming et al. 2020). In contrast with other substances associated with involvement with correctional services, e.g. opioids (Chen et al. 2022) and alcohol (Akbar et al. 2018), there are no accepted pharmacological treatments for methamphetamine use (Siefried et al. 2020). The only existing treatments are psychosocial and behavioral – a systematic review demonstrated CBT and contingency management to be effective in reducing use of the drug (AshaRani et al. 2020), and therapeutic communities have also been demonstrated to confer therapeutic benefits in correctional (Joe et al. 2010) and community settings (Šefránek & Miovský 2017). However, there may be barriers to accessing treatment for methamphetamine use, including financial costs and lack of available places in programs (Cumming et al. 2016).

A significant barrier to accessing both community and correctional in-person SUD services since March 2020 has been the COVID-19 pandemic. Krebs and colleagues (2021) examined the impact of the pandemic on corrections agencies across Ohio – 75% of respondents reported restrictions that impacted rehabilitative programming, including SUD treatment. Massachusetts (Donelan et al. 2021) and New York State (Wang et al. 2021) implemented telehealth approaches to provide medications for opioid use disorders during the pandemic. Such technology-mediated approaches have been explored for the treatment of methamphetamine use in community settings (Rubenis et al. 2021) – this review not find significant reductions in methamphetamines use and suggested this could be due to app design features such as whether content was personalized, and participant characteristics such as personality style.

### The potential of digital cognitive behavioral therapy in treating methamphetamine use

Digital technology can also be used to deliver CBT for SUD (e.g. Carroll et al. 2009; Kay-Lambkin et al. 2009), although these programs have largely been delivered in community settings due to the security challenges that exist in prisons and jails. The only digital CBT intervention for SUD that has been delivered in secure settings is ‘Breaking Free from Substance Abuse’, which has been available in UK prisons since 2015 (Davies et al. 2017; Elison et al. 2015). The program addresses dependence to multiple substances and also has demonstrated efficacy in community treatment services (e.g. Elison, Jones, et al. 2017; Elison, Ward, et al. 2017), for people using alcohol (Ward et al. 2019), cannabis (Elison-Davies, Wardell, et al. 2021) and opiates (Elison-Davies, Märtens, et al. 2021). The program has demonstrated user acceptability (e.g. Dugdale et al. 2017; Elison et al. 2015) and characteristics of individuals accessing the program via UK treatment services have also been reported (e.g. Elison, Jones, et al. 2017; Elison-Davies, Hayhurst, et al. 2021).

**Fig. 1:**
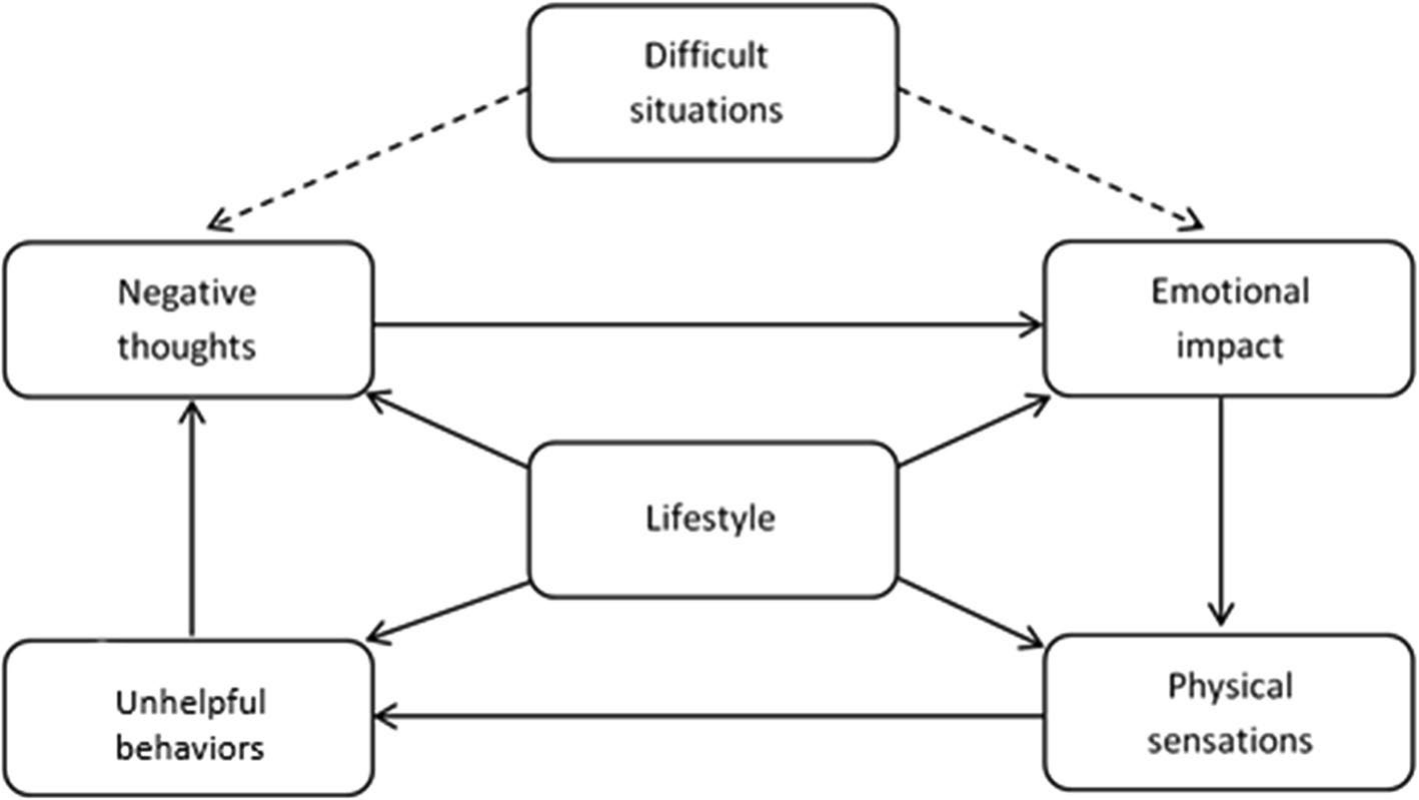
The lifestyle balance model

The program is modular and targets the psychological and lifestyle factors that underlie SUDs. When an individual first uses it they complete a baseline assessment, which includes the ‘Recovery Progression Measure’ (RPM: Elison et al. 2016) – the RPM measures levels of functioning in six biopsychosocial domains (see Fig. 1). The program uses data provided by the individual when they complete the RPM to populate a visual depiction of a theoretical CBT framework, the ‘Lifestyle Balance Model’ (LBM: Davies et al. 2015), which shows them the extent to which they may be experiencing impairments in the six domains of functioning measured by the RPM and represented in the LBM. The program achieves this by color coding each of the six components of the LBM – red domains represent significant impairment, amber moderate impairment, and green low/no impairment. The LBM is based on the five-factor model used in CBT (Williams & Garland 2002) and incorporates principles contained in the RNR model, in terms of focusing on the interplay between an individual’s cognitions, emotions, behaviors and lifestyle factors (Bonta & Andrews 2007).

Within the program are 12 evidence-based ‘behavioral change techniques’ (BCTs: Dugdale et al. 2016; Michie et al. 2013) that are effective in reducing substance use and biopsychosocial impairment – these 12 BCTs are located within the six components of the LBM. Each component of the LBM contains two BCTs – a psychoeducational ‘Information Strategy’ and a skills-building interactive ‘Action Strategy’. In order to complete a component of the LBM, an individual has to complete both the Information and Action Strategy associated with that component. Table 1 provides a full description of the BCTs contained in the program.

**Table 1:** The ‘behavioral change techniques’ (BCTs) contained within Breaking Free from Substance Abuse

Content in Breaking Free Online	Description of strategy	Therapeutic approaches underpinning strategies	BCT taxonomy (V1) techniques
Baseline and progress check assessments	Monitoring behavior to provide feedback about progress towards goals; Encouraging new behaviors via positive feedback	Goal setting; self-monitoring	Self-monitoring of behavior; Feedback on outcome(s) of behavior
Lifestyle Balance Model	Generic formulation; Idiosyncratic formulation; Personalized feedback; Case formulation – understanding the links between situations, thoughts, emotions, behaviors, physical sensations and lifestyle	Node-link mapping (International Treatment Effectiveness Project - ITEP); Cognitive-behavioral therapy (CBT)	Information about antecedents; Information about health consequences; Salience of consequences; Information about social and environmental consequences; Information about emotional consequences
Difficult situations domain of LBM	Assessment; Self-monitoring; Standardized measures; Psychoeducation on impact of problematic situations; Intervention to help people in distress access support; Recognize–avoid–cope; Relapse prevention for coping with environmental/situational/emotional triggers; Creating action plans on how to avoid or cope in high-risk situations	Psychoeducation; Guided self-help; Relapse prevention; Refusal skills	Social support (unspecified); Reduce negative emotions; Problem solving; Action planning; Instruction on how to perform the behavior; Behavioral practice/rehearsal; Behavior substitution; Avoidance/reducing exposure to cues for the behavior; Goal setting
Negative thoughts domain of LBM	Psychoeducation on impact on negative thoughts; Mind traps; Cognitive restructuring; Challenging thoughts that may be unhelpful	Psychoeducation; Guided self-help; Node-link mapping (International Treatment Effectiveness Project - ITEP); Cognitive-behavioral therapy (CBT)	Information about antecedents; Information about health consequences; Salience of consequences; Information about social and environmental consequences; Information about emotional consequences; Re-attribution; Framing-reframing
Emotions domain of LBM	Psychoeducation on impact on emotions; Attention narrowing; Attention switching; Emotional regulation; Recognizing/understanding/normalizing emotions; Developing more appropriate coping strategies	Psychoeducation; Guided self-help; Coping strategy enhancement (CSE); Mindfulness-based cognitive therapy	Information about antecedents; Information about health consequences; Salience of consequences; Information about social and environmental consequences; Information about emotional consequences; Behavioral practice/rehearsal; Reducing negative emotions; Problem solving; Social support (unspecified); Behavioral practice/rehearsal; Distraction
Physical sensations domain of LBM	Psychoeducation on impact of physical sensations; Urge surfing; Body scanning; Relapse prevention-based techniques	Psychoeducation; Guided self-help; Mindfulness-based cognitive therapy	Information about antecedents; Information about health consequences; Salience of consequences; Information about social and environmental consequences; Information about emotional consequences; Instruction on how to perform a behavior; Behavioral practice/rehearsal; Reducing negative emotions
Unhelpful behaviors domain of LBM	Psychoeducation on impact of destructive behaviors; Activity scheduling; Behavioral activation; Encouraging new behaviors via positive feedback; Increasing activity to increase energy levels and relieve boredom	Psychoeducation; Guided self-help; Cognitive-behavioral therapy (CBT)	Information about antecedents; Information about health consequences; Salience of consequences; Information about social and environmental consequences; Information about emotional consequences; Non-specific reward; Non-specific incentive; Reward approximation; Rewarding completion; Goal setting (behavior); Action planning
Lifestyle domain of LBM	Psychoeducation on impact of lifestyle; Creating SMART goals for recovery; Goal setting; Increasing treatment engagement and retention; Increasing readiness to change behavior	Psychoeducation; Guided self-help; Motivational enhancement therapy (MET); Implementation intentions	Goal setting (behavior); Problem solving; Goal setting (outcome); Action planning; Non-specific reward; Focusing on past success

The program provides tailoring advice and recommends that users focus on first completing the components of the program that will allow them to address the areas of functioning where they are experiencing the most impairment, i.e. the areas of the LBM that are colored red – research indicates people follow this tailoring advice (Elison, Jones, et al. 2017). A ‘dose response’ has also been demonstrated, which suggests the more components of the program someone completes, the better their outcomes (Elison-Davies, Wardell, et al. 2021). Significant reductions in substance dependence, depression and anxiety, and biopsychosocial impairment, and improvements in quality of life, have been demonstrated for individuals who engage with the program in UK community settings (Elison et al. 2014; Elison, Ward, et al. 2017) and prisons (Davies et al. 2017; Elison et al. 2015).

### Delivery of Breaking Free from Substance Abuse across Ohio Department of Rehabilitation and Correction

Since May 2020 Breaking Free from Substance Abuse has been available via secure tablets to prison residents across Ohio Department of Rehabilitation and Correction (ODRC). ODRC have had access to secure devices for some time – these devices were originally introduced to deliver educational content. Following rigorous security checks, Breaking Free from Substance Abuse was added as additional content to the devices at the start of the COVID-19 pandemic when in-person rehabilitative programs became unavailable. The program was rolled-out across all 28 adult facilities in a phased manner between May and November 2020. During this period, the developers of the program trained over 100 staff across ODRC so they could support residents using the it.

### Aims

Delivery of the program across ODRC has allowed, for the first time, data to be collected from incarcerated individuals in the US engaging with this digital CBT for SUD. This study reports data from methamphetamine users given the high rates of psychosocial risk factors experienced by this population, the significant links between methamphetamine use and incarceration (Cumming et al. 2020) and the lack of pharmacological treatments for use of the drug (Siefried et al. 2020). To date, all published data from this digital CBT program has come from individuals engaging with it via UK treatment services (e.g. Elison-Davies, Hayhurst, et al. 2021), where methamphetamine is not a commonly used drug – a recent government report from 2020 to 2021 showed that only 505 members of the UK treatment population reported methamphetamine use (Office for Health Improvement and Disparities 2021). Therefore, this study also provides the first opportunity to report data from people engaging with the program for methamphetamine use.

This study uses a within-subjects design to examine changes over time on a number of measures collected at baseline and post-engagement. These data are used to examine: i) baseline psychosocial risk factors experienced by Ohio prison residents, ii) changes in their scores on measures of substance dependence, mental health, quality of life and biopsychosocial impairment when engaging with the program and iii) whether baseline psychosocial risk factors are associated with these changes. Additionally, iv) patterns of engagement with components of the program, and the extent to which program engagement is associated with changes in scores on the measures used, is also reported.

## Methods

### Design

Quantitative within-subjects, non-randomized observational study to examine associations between psychosocial risk factors, and engagement with a digital CBT program for SUD, with changes in scores on a range of measures, for prison residents who used the program to address their methamphetamine use during the COVID-19 pandemic.

### Participants

Two thousand one hundred and eighty-seven ODRC residents who engaged with digital CBT to address their methamphetamine use between May 2020 and September 2021.

### Procedure

Approval to analyze data provided by participants was granted by the ODRC Human Subjects Research Review Committee on May 3rd 2021. Residents who had been referred to prison SUD services were offered the digital CBT program whilst in-person services were unavailable due to COVID-19 restrictions. The program was made available via secure tablet computers – participants could sign-in to the tablet using their own unique login credentials to access the program, which was available via a link on the tablet landing page. Before participants could activate an account on the program they had to agree to a ‘Privacy and Cookies Policy’ and ‘End User License Agreement’. After account activation all participants were required to complete a baseline assessment containing several standardized measures:

- *Severity of Dependence Scale* (SDS: Gossop et al. 1997): 5-item scale measuring severity of substance dependence (e.g., cravings and substance-related cognitions). Internal reliability: *α* = .81–.90; test-retest reliability *ICC* = .89.
- *Patient Health Questionnaire-4* (PHQ-4: Kroenke et al. 2009): 4-item scale measuring severity of depression and anxiety. Threshold scores on the PHQ are 0–3 no depression/anxiety, 3–5 ‘mild’, 6–8 ‘moderate’ and 9–12 ‘severe’. Internal reliability, *α* = .81.
- *Five items (1, 2, 17, 18, 20) from the World Health Organization Quality of Life measure* (WHOQoL-BREF: Skevington et al. 2004). Items selected were generic enough to measure general quality of life as opposed to specific aspects of quality of life. Internal reliability of these five items, *α* = .84.
- *Recovery Progression Measure* (RPM: Elison et al. 2016; Elison, Dugdale, et al. 2017): 36-item scale measuring functioning in the six domains of biopsychosocial functioning represented by the components of the LBM (see Fig. 1). Within each of the six RPM domains there are five dichotomous ‘yes/no’ items measuring presence or absence of specific biopsychosocial difficulties within that domain, and an 11-point Likert ‘impact scale’ assessing level of severity of impairment in that domain. Internal consistency, *α* = .89; test-retest reliability, *ICC* = .73.
- In addition to the standardized measures the baseline assessment also contained the following:

- *Demographic items:* age, gender, and ethnicity.
- *Questions about what substances each participant was experiencing difficulties with –* this information was also used to determine whether participants were poly-substance users.

The program’s backend database also automatically captured program engagement data: i) whether participants completed a follow-up assessment, and ii) which BCTs they had completed.

After baseline assessment completion, participants were provided with access to the program and engaged with it in a self-directed manner. Each participant’s account was available to them for 12-months and they could choose to engage with the program for as long as they wanted to during this period. The program prompts users to complete mandatory ‘Progress Check’ assessments every two weeks – people can also choose to complete Progress Check assessments more frequently. Data from the Progress Checks allow an individual to monitor their progress as they work through the program via a personalized dashboard.

### Data analysis

The majority or variables reasonably approximated a non-normal distribution (skewness > 1, kurtosis > 2, Shapiro-Wilks < .05). Kruskall-Wallis tests were used to compare baseline assessment measure scores of i) participants who had completed a Progress Check and ii) those that had not completed a Progress Check to ascertain whether differences between these groups might explain why some participants completed a Progress Check assessment and some did not. For participants that did complete a Progress Check assessment, comparisons of scores on the assessment measures included from baseline to most recent Progress Check data were conducted using repeated-measure Mann-Whitney U tests and Cohen’s effect sizes. Whilst controlling for the confounding effects of baseline scores on outcomes on the assessment measures included, linear regressions were used to examine associations between psychosocial risk factors reported at baseline and outcomes scores obtained from most recent Progress Check assessment – this was done in order to ascertain whether baseline psychosocial risk factors might predict outcomes. Associations between whether or not each of the six components in the program had been completed and outcomes scores obtained from most recent Progress Check were also examined using linear regressions – this was done to ascertain whether completion of specific components of the program might predict outcomes. Finally, linear regressions were also used to examine associations between the number of components completed and outcomes on each of the assessment measures included, in order to determine whether there may be a ‘dosage effect’. Due to the number of comparisons in the analyses, a more conservative significance level of *p* = .01 was adopted.

## Results

### Sociodemographic characteristics

The largest age group was 25–34 years (896, 41%), followed by 35–44 years (766, 35%), 45–54 years (236, 11%), 18–24 years (223, 10%), 55–64 years (48, 2%), with the smallest age group being 65 years and over (6, 0.4%) – 12 (0.6%) participants did not provide their age. There were approximately twice as many male as female participants – 1416 (65%) reported identifying as male and 734 (33.5%) reported identifying as female. A total of 23 (1%) participants identified as belonging to a different gender group and 14 participants (0.5%) did not provide their gender. The largest ethnic group was White Americans (1854, 85%), followed by those having mixed heritage (121, 5.5%), Black or African Americans (86, 4%), Hispanic or Latino Americans (56, 2.6%), American Indian or Alaskan Natives (24, 1%), Asian Americans (7, 0.3%), with the smallest ethnic group being Native Hawaiian and other Pacific Islanders (3, 0.1%). A total of 36 (1.5%) participants did not provide their ethnicity.

### Psychosocial risk factors

A total of 1427 (65%) participants reported engaging with the program to address their methamphetamine use only, 695 (32%) to address their use of methamphetamine plus one other substance, and 65 (3%) to address their use of methamphetamine plus two other substances. 1511 (69%) participants reported moderate to severe substance dependence (SDS total score ≥ 4) and 1259 (58%) participants reported moderate to severe depression/anxiety (PHQ-4 total score ≥ 6). Participants reported a number of risk factors measured by the RPM including difficult close relationships (1358, 62%), interpersonal conflict (1040, 48%), risk taking behaviors (1176, 54%), aggression (1137, 52%) and paranoid thoughts (989, 45%). Lifestyle difficulties were also reported including poor health (989, 45%), problems with work and education (625, 29%), unstable accommodation (1062, 49%) and financial difficulties (1127, 52%).

### Comparison of baseline and Progress check assessment data

Of the 2187 participants who completed a baseline assessment, 1150 (53%) completed a Progress Check assessment – of those that did complete a Progress Check assessment, the mean number of assessments completed was four. The mean number of days between baseline assessment and most recent Progress Check assessment was 77.32 days (*SD* 96.20) or 11.05 weeks. No baseline differences were found between those participants who did complete a Progress Check assessment and those that did not (see Table 2).

**Table 2:** Between-subject difference in baseline assessment scores for methamphetamine users who did and did not complete a Progress Check assessment when engaging with Breaking Free from Substance Abuse

Outcome Measure	No Progress Check completed Mean (*SD*)	Progress Check completed Mean (*SD*)	F	*p*	d
SDS	7.50 (4.58)	7.20 (4.56)	−1.502	.133	0.07
PHQ	6.29 (3.55)	6.39 (3.63)	0.583	.560	0.03
WHOQoL-BREF	15.58 (3.87)	15.70 (3.75)	0.603	.547	0.03
RPM	33.30 (13.25)	33.09 (13.24)	−0.229	.819	0.02

For those participants that did complete a Progress Check, significant changes were found in scores across all measures (see Table 3), including reductions in methamphetamine dependence (SDS), depression/anxiety (PHQ), and biopsychosocial impairment (RPM), and improvements in quality of life (WHOQoL-BREF) (all *p* < .001). Effect size calculations demonstrated small effects for reductions in substance dependence (SDS: *d* = .45) and depression/anxiety (PHQ-4: *d* = 0.49), and improvements in quality of life (WHOQoL-BREF: *d* = 0.47), and a medium effect for reductions in biopsychosocial impairment (RPM: *d* = 0.54).

**Table 3:** Within-subject outcomes for participants engaging with Breaking Free from Substance Abuse in order to address their methamphetamine use

Outcome Measure	Mean at baseline (*SD*)	Mean at post-treatment (*SD*)	F	*p*	d
SDS	7.20 (4.56)	5.24 (4.83)	−12.10	<.001	0.45
PHQ	6.39 (3.63)	4.46 (4.03)	−14.66	< .001	0.49
WHOQoL-BREF	15.70 (3.75)	17.70 (4.99)	13.36	< .001	0.47
RPM	33.09 (13.24)	25.00 (17.26)	−14.12	< .001	0.54

### Associations between psychosocial risk factors and outcomes

Whilst controlling for baseline scores, a number of psychosocial risk factors appeared to predict scores at most recent Progress Check assessment (see Table 4). Results of the linear regressions conducted indicated that there was a collective significant effect between each of the risk factors included in the analysis on scores at most recent Progress Check: substance dependence (SDS: *F* (12,1136) = 18.700, *p* < .001, *R*^*2*^ = 0.165), depression/anxiety (PHQ-4: *F*(12,1137) = 24.721, *p* <,001, *R*^*2*^ = 0.207), quality of life (WHOQoL-BREF: *F*(12,1137) = 12.233, *p* < .001, *R*^*2*^ = 0.114) and biopsychosocial impairment (RPM: *F*(12,1137) = 12.537, *p* < .001, *R*^*2*^ = 0.117).

**Table 4:** Associations between baseline psychosocial risk factors and outcomes for individuals engaging with Breaking Free from Substance Abuse in order to address their methamphetamine use

Outcome measure	Baseline psychosocial criminogenic risk factors	β	t	p	95% CI		Overall regression model (association between outcomes and baseline participant characteristics)			
					Lower	Upper	R^2^	F	df	*p*
SDS	Total SDS score	.372	12.775	<.001	.334	.455				
	Polysubstance user	.033	1.192	.233	−.213	.874				
	Total PHQ-4 score	.028	0.861	.389	−.048	.123				
	RPM item: Paranoid thoughts	.000	0.006	.995	−.744	.749				
	RPM item: Difficulties in close relationships	.033	1.021	.308	−.302	.956				
	RPM item: Conflict with other people	.046	1.484	.138	−.144	1.038				
	RPM item: Poor health	.015	0.513	.608	−.434	.742	0.165	18.700	12, 1136	< .001
	RPM item: Problems with work/education	−.035	−1.172	.242	−.999	.252				
	RPM item: Unstable accommodation	−.013	−0.425	.671	−.727	.468				
	RPM item: Financial difficulties	.023	0.757	.449	−.350	.790				
	RPM item: Risk taking behaviors	−.003	−0.087	.931	−.618	.566				
	RPM item: Aggression	−.005	−0.171	.864	−.649	.545				
PHQ-4	Total SDS score	.046	1.607	.108	−.009	.090				
	Polysubstance user	.013	0.485	.628	−.333	.551				
	Total PHQ-4 score	.365	11.473	<.001	.337	.475				
	RPM item: Paranoid thoughts	−.047	−1.582	.114	−1.097	.118				
	RPM item: Difficulties in close relationships	.028	0.883	.377	−.281	.742				
	RPM item: Conflict with other people	.073	2.409	.016	.109	1.070				
	RPM item: Poor health	−.006	−0.195	.846	−.525	.430	0.207	24.721	12, 1137	< .001
	RPM item: Problems with work/education	.045	1.522	.128	−.114	.903				
	RPM item: Unstable accommodation	−.001	−0.047	.963	−.497	.474				
	RPM item: Financial difficulties	.011	0.376	.707	−.375	.552				
	RPM item: Risk taking behaviors	.040	1.313	.190	−.159	.803				
	RPM item: Aggression	.059	1.936	.053	−.006	.963				
WHOQoL-BREF	Total SDS score	.041	1.365	.172	−.020	.109				
	Polysubstance user	−.050	−1.775	.076	−1.100	.055				
	Total PHQ-4 score	−.208	−6.195	<.001	−.377	−.196				
	RPM item: Paranoid thoughts	−.025	−0.783	.434	−1.111	.477				
	RPM item: Difficulties in close relationships	−.037	−1.129	.259	−1.054	.284				
	RPM item: Conflict with other people	−.063	−1.950	.051	−1.252	.004				
	RPM item: Poor health	−.046	−1.535	.125	−1.114	.136	0.114	12.233	12, 1137	< .001
	RPM item: Problems with work/education	−.075	−2.416	.016	−1.484	−.154				
	RPM item: Unstable accommodation	−.013	−0.396	.692	−.763	.507				
	RPM item: Financial difficulties	−.033	−1.069	.285	−.936	.276				
	RPM item: Risk taking behaviors	−.039	−1.230	.219	−1.023	.235				
	RPM item: Aggression	.007	0.203	.839	−.568	.699				
RPM	Total SDS score	.042	1.414	.158	−.062	.383				
	Polysubstance user	.028	0.990	.322	−.988	3.002				
	Total PHQ-4 score	.201	5.996	<.001	.645	1.272				
	RPM item: Paranoid thoughts	.034	1.077	.282	−1.237	4.248				
	RPM item: Difficulties in close relationships	−.006	−0.167	.867	−2.506	2.113				
	RPM item: Conflict with other people	.051	1.608	.108	−.391	3.945				
	RPM item: Poor health	.000	−0.003	.998	−2.161	2.155	0.117	12.537	12, 1137	< .001
	RPM item: Problems with work/education	.047	1.525	.128	−.512	4.082				
	RPM item: Unstable accommodation	.010	0.303	.762	−1.854	2.530				
	RPM item: Financial difficulties	.026	0.847	.397	−1.189	2.997				
	RPM item: Risk taking behaviors	.062	1.932	.054	−.034	4.311				
	RPM item: Aggression	.061	1.899	.058	−.070	4.308				

Individual risk factors appeared to be significant predictors of scores at most recent Progress Check. Conflict with others was significantly positively associated with depression/anxiety scores (PHQ-4: *t* = 2.409, *p* = .016) and problems with work or education was significantly negatively associated with quality of life scores (WHOQoL-BREF: *t* = − 2.416, *p* < .001). Additionally, baseline depression/anxiety was significantly negatively associated with quality of life scores (WHOQoL-BREF: *t* = − 6.195, *p* < .001) and significantly positively associated with biopsychosocial impairment scores (RPM: *t* = 5.996, *p* < .001).

### Associations between program engagement and outcomes

Program engagement was also examined, including the number of components completed (out of a possible maximum of six), and which individual ‘Information Strategies’ and ‘Action Strategies’ (the 12 BCTs in the program) were completed. In order to complete a component of the program, an individual has to complete both the Information Strategy and corresponding Action Strategy contained within a component. The mean number of components completed by participants who provided Progress Check assessment data was 2.44 (*SD* 2.63), and 0.17 (*SD* 0.73) for those who did not provide Progress Check data. Table 5 demonstrates the numbers of both Progress Check assessment completers and non-completers who completed each of the 12 individual BCTs included in the six components of the program (the six Information Strategies and the six Action Strategies).

**Table 5:** Numbers of Progress Check assessment completers and non-completers who completed each of the 12 main behavioral change techniques in Breaking Free from Substance Abuse

Behavioral change techniques		Progress Check assessment completed			No Progress Check assessment completed		
		N	%	Rank	N	%	Rank
Information Strategies	Physical Sensations	482	41.9	5	32	3.1	5
	Difficult Situations	646	56.2	1	89	8.6	1
	Negative Thoughts	575	50	2	61	5.9	2
	Emotions	530	46.1	3	36	3.5	4
	Unhelpful Behaviors	478	41.6	6	29	2.8	6
	Lifestyle	499	43.4	4	40	3.9	3
Action Strategies	Physical Sensations	550	47.8	5	53	5.1	5
	Difficult Situations	689	59.9	1	104	10	1
	Negative Thoughts	673	58.5	2	88	8.5	2
	Emotions	647	56.3	3	80	7.7	3
	Unhelpful Behaviors	537	46.7	6	38	3.7	6
	Lifestyle	614	53.4	4	75	7.2	4

Whilst controlling for baseline scores, completion of the six components in the program appeared to predict scores at most recent Progress Check assessment (see Table 6). Results of the linear regressions conducted indicated that there was a collective significant effect between completion of program components on each of the scores at most recent Progress Check: substance dependence (SDS: F(7,1141) = 37.361, *p* < .001, R^2^ = 0.432), depression/anxiety (PHQ-4: F(7,1142) = 61.187, *p* <,001, R^2^ = 0.273), quality of life (WHOQoL-BREF: F(7,1142) = 59.729, *p* < .001, R^2^ = 0.268) and biopsychosocial impairment (RPM: F(7,1142) = 66.206, *p* < .001, R^2^ = 0.289) (see Table 6).

**Table 6:** Associations between the total number of components of Breaking Free from Substance Abuse completed and outcomes, for individuals engaging with the program in order to address their methamphetamine use

Outcome measure	Breaking Free from Substance use components	β	t	p	95% CI		Overall regression model (association between outcomes and baseline participant characteristics)			
					Lower	Upper	R^2^	F	df	*p*
SDS	Physical Sensations	−.035	−0.614	.540	−1.485	.777				
	Difficult Situations	−.011	−0.260	.795	−.936	.717				
	Negative Thoughts	−.010	−0.199	.843	−1.035	.845	0.432	37.361	7, 1141	< .001
	Emotions	.003	0.058	.954	−.996	1.057				
	Unhelpful Behaviors	−.117	−1.998	.046	−2.340	−.021				
	Lifestyle	−.011	−0.208	.835	−1.172	.947				
PHQ-4	Physical Sensations	−.099	−1.809	.071	−1.715	.070				
	Difficult Situations	−.034	−0.820	.412	−.924	.379				
	Negative Thoughts	.044	0.945	.345	−.384	1.099	0.273	61.187	7, 1142	< .001
	Emotions	.026	0.508	.612	−.601	1.021				
	Unhelpful Behaviors	−.118	−2.119	.034	−1.902	−.073				
	Lifestyle	−.137	−2.659	.008	−1.970	−.297				
WHOQoL-BREF	Physical Sensations	.094	1.714	.087	−.140	2.075				
	Difficult Situations	.059	1.429	.153	−.220	1.400				
	Negative Thoughts	−.034	−0.723	.470	−1.260	.582	0.268	59.729	7, 1142	< .001
	Emotions	−.019	−0.381	.703	−1.200	.810				
	Unhelpful Behaviors	.096	1.729	.084	−.135	2.136				
	Lifestyle	.151	2.934	.003	.514	2.589				
RPM	Physical Sensations	−.037	−0.692	.489	−5.108	2.445				
	Difficult Situations	−.110	−2.693	.007	−6.552	−1.029				
	Negative Thoughts	.074	1.611	.107	−.561	5.716	0.289	66.206	7, 1142	< .001
	Emotions	−.047	−0.947	.344	−5.083	1.773				
	Unhelpful Behaviors	−.178	−3.244	.001	−10.269	−2.528				
	Lifestyle	−.163	−3.201	.001	−9.306	−2.233				

Completion of individual program components appeared to significantly predict some scores at most recent Progress Check. Completion of the Difficult Situation component was significantly negatively associated with biopsychosocial impairment scores (RPM: *t* = − 2.693, *p* = .007). Completion of the Unhelpful Behaviors component was significantly negatively associated with substance dependence (SDS: *t* = − 1.998, *p* = .046) depression/anxiety (PHQ-4: *t* = − 2.119, *p* = .034) and biopsychosocial impairment scores (RPM: *t* = − 3.244, *p* = .001). Completion of the Lifestyle component was significantly negatively associated with depression/anxiety (PHQ-4: *t* = − 2.659, *p* = .008) and biopsychosocial impairment scores (RPM: *t* = − 3.201, *p* = .001), and significantly positively associated with quality of life scores (WHOQoL-BREF: *t* = 2.934, *p* = .003).

A dose response was also identified, with the total number of program components completed being significantly negatively associated with substance dependence (SDS: F(2,1146) = 130,063, *p* < .001, R^2^ = 0.185), depression/anxiety (PHQ-4: F(2,1147) = 202.043, *p* <,001, R^2^ = 0.261) and biopsychosocial impairment scores at most recent Progress Check (RPM: F(2,1147) = 207.762, *p* < .001, R2 = 0.266), and positively associated with quality of life scores (WHOQoL-BREF: F(2,1147) = 205.271, *p* < .001, R^2^ = 0.0.264).

## Discussion

This study reports data from 2187 methamphetamine-involved Ohio prison residents who engaged with a digital CBT program for SUD, ‘Breaking Free from Substance Abuse’, during the COVID-19 pandemic. Although there were twice as many male as female participants, females were over-represented in relation to Ohio’s prison population – there are only two women’s facilities in the state compared to 23 men’s facilities. This might demonstrate that women with SUD may be more receptive to digital CBT programs and deserves further investigation.

Participants reported a range of psychosocial risk factors that are common amongst methamphetamine uses, including interpersonal conflict (Dadhe & Bettman 2019; Maltman et al. 2020), mental health difficulties including paranoia (Dadhe & Bettman 2019; McKetin et al. 2019), impulsive and aggressive behaviors (Lanesman et al. 2019) and unstable accommodation and homelessness (Jones et al. 2020; Moxley et al. 2020). These difficulties have also been identified in the literature as *criminogenic* risk factors (Andrews et al. 2006) and are commonly experienced by incarcerated individuals who use methamphetamine (Semple et al. 2008).

Half of participants completed both a baseline assessment and a Progress Check assessment – significant reductions were found in scores for severity of substance dependence, depression/anxiety and biopsychosocial functioning impairment, and a significant increase in quality of life was demonstrated. Effect sizes for changes in substance dependence severity, depression/anxiety, and quality of life were small, and the effect size for changes in biopsychosocial functioning impairment was medium. Studies of other CBT interventions for methamphetamine use (AshaRani et al. 2020; Lee & Rawson 2008), and previous studies of this digital CBT program with UK prison populations (Davies et al. 2017; Elison et al. 2015), have obtained similar findings.

Psychosocial risk factors reported were significantly associated with most recent Progress Check assessment scores. Interpersonal conflict was significantly associated with poorer mental health and quality of life outcomes, and more severe depression/anxiety was significantly associated with poorer quality of life and biopsychosocial functioning outcomes. Research has demonstrated that such psychosocial risk factors may predict treatment outcomes in methamphetamine users, for example, interpersonal/relationship instability (Brecht & Herbeck 2014) and mental health difficulties (McKetin et al. 2018).

Participants who completed a Progress Check assessment completed more of the BCTs and components in the program that those that did not complete a Progress Check. For both Progress Check completers and non-completers, the Action and Information Strategies completed by the most participants were those in the Difficult Situations component of the program, and strategies completed by the least participants were those in the Unhelpful Behaviors component of the program. The Difficult Situations component supports individuals to develop skills to avoid and cope with difficult or risky situations – having these skills is particularly important in a prison environment where there may be multiple risks. However, the Unhelpful Behaviors component supports individuals to plan positive activities for each day of the week – the constraints of the prison environment could prevent incarcerated people from being able to identify activities that they can feasibly enact.

Completion of some components within the program was significantly associated with scores at most recent Progress Check. Completion of the Difficult Situations component was significantly associated with better biopsychosocial functioning scores, and completion of the Unhelpful Behaviors component was significantly associated with lower substance dependence, and better mental health and biopsychosocial functioning scores. Completion of the Lifestyle component was significantly associated with better mental health, biopsychosocial functioning and quality of life scores. These findings indicate that interventions for methamphetamine use should incorporate self-efficacy and coping skills development components (Moos 2007), in addition to clinical approaches to support emotional regulation (Kang et al. 2019) and positive lifestyle changes (Juel et al. 2017). The dose response identified in previous studies of this specific digital CBT program (Elison, Jones, et al. 2017; Elison-Davies, Wardell, et al. 2021), and other digital CBT for SUD studies (Mattila et al. 2016), was replicated in this study.

Findings suggest that greater clinical complexity at baseline might mean some individuals may benefit from support to enhance their level of engagement with the components of the program, in order to ensure they can experience optimal clinical benefits. When this digital CBT program is delivered alongside practitioner support, this can enhance retention and improve outcomes (Elison et al. 2014; Elison, Ward, et al. 2017). The literature demonstrates that individuals with SUD may be at risk of dropping out of treatment if they are not appropriately supported (Şimşek et al. 2019). Additionally, the literature around digital CBT for depression and anxiety demonstrates greater adherence and better outcomes when such programs are delivered alongside practitioner support (Andersson et al. 2019). Similar findings have been obtained for digital CBT for alcohol use disorder (Sundström et al. 2016), although there are gaps in the literature around whether practitioner guided or unguided digital CBT is most appropriate for other kinds of substance use disorders (Boumparis et al. 2019).

Although this study demonstrates that digital technologies can enhance rehabilitative programming, traditional in-person delivery may still be preferred by prison residents and staff. Introduction of such technologies can sometimes be perceived by staff as being disruptive as they may not feel they have the capacity to become familiar with new technologies (Davies et al. 2017). There may also be concerns around the security implications of providing digital technologies in secure settings – there have been examples of incarcerated people using digital technologies for nefarious means. However, work can be done to ensure rehabilitative technologies undergo thorough security checks before they are introduced into correctional settings, as was the case during the introduction of Breaking Free from Substance Use across ODRC.

As digital rehabilitative programming becomes more common, many of the benefits people experience from in-person support might be lost – however, digital programs need not eradicate the role of staff in program delivery. Many digital CBT programs are designed to be delivered as self-directed interventions and also ‘computer-assisted therapies’, with users being supported to engage with program content by staff. Additionally, digital programs are not necessarily designed to replace in-person support but augment it, especially during times when in-person support may be limited.

Had Breaking Free from Substance Use been introduced across ODRC before the pandemic it is not known whether the same amount of engagement would have been seen. However, the interactive nature of digital programs may be appealing to people in prisons and jails who may be bored or may have difficulties with concentration – work completed by the authors years before the COVID-19 pandemic indicated that even in non-pandemic times this treatment modality can be well received by incarcerated individuals (Davies et al. 2017; Elison et al. 2015). And regardless, it looks likely that health service delivery, and rehabilitation programming delivery, has been changed for the long-term as a result of the heavy reliance on digital technology during the COVID-19 pandemic.

### Limitations

Limitations include that participants were self-selecting and a comparison group was not included given this study was an exploratory observational study, not a randomized controlled trial (RCT). Only half of participants completed a Progress Check assessment – the reasons for this could not be determined from data available. Attrition rates in digital intervention studies have been identified as a problem (Eysenbach 2005), with a recent review finding that even in highly controlled RCTs, the average attrition rate is 48% (Torous et al. 2020) – attrition in studies with SUD populations can also be high (Radtke et al. 2017). Additionally, it is difficult to determine whether changes in assessment scores were due to the clinical impact of the program or because some participants had higher levels of motivation and readiness to change. Therefore, determining effectiveness of the program for methamphetamine users via a RCT is an appropriate next step – a RCT could also build in longitudinal follow-up to explore how people fare when they return back to the community from prison.

## Conclusions

This study reports data from US prison residents engaging with a digital CBT program for SUD. This study identified high rates of psychosocial risk factors commonly associated with methamphetamine use, and that these risk factors were associated with biopsychosocial changes experienced by people engaging with this program. Despite the clinical complexity of the group, they experienced significant reductions in substance dependence, depression/anxiety, and biopsychosocial impairment, and significant improvements in quality of life. Program engagement was associated with these changes and a dose response was identified, indicating that some prison residents might benefit from support when engaging with digital programs such as Breaking Free from Substance Abuse.

## Data Availability

The datasets generated and/or analysed during the current study are not publicly available for data privacy and security reasons. However, data can be made available upon request to the Corresponding Author for specific studies that have been reviewed and approved by a Research Ethics Board/Committee.

## Abbreviations

BCT: behavioral change technique
CBT: cognitive behavioral therapy
ODRC: Ohio Department of Rehabilitation and Corrections
PHQ-4: Patient Health Questionnaire-4
RPM: Recovery Progression Measure
SDS: Severity of Dependence Scale
SUD: substance use disorder
UK: United Kingdom
US: United States
WHOQoL-BREF: World Health Organization Quality of Life Scale
